# A Scalable and Robust Chloroplast Genotyping Solution: Development and Application of SNP and InDel Markers in the Maize Chloroplast Genome

**DOI:** 10.3390/genes15030293

**Published:** 2024-02-25

**Authors:** Rui Wang, Yang Yang, Hongli Tian, Hongmei Yi, Liwen Xu, Yuanda Lv, Jianrong Ge, Yikun Zhao, Lu Wang, Shiliang Zhou, Fengge Wang

**Affiliations:** 1 Maize Research Institute, Beijing Key Laboratory of Maize DNA Fingerprinting and Molecular Breeding, Key Laboratory of Crop DNA Fingerprinting Innovation and Utilization (Co-construction by Ministry and Province), Beijing Academy of Agricultural and Forest Sciences (BAAFS), Beijing 100097, China; skywangr@126.com (R.W.); caurwx@163.com (Y.Y.); tianhongli9963@163.com (H.T.); yhmmaize@163.com (H.Y.); xulw0408@126.com (L.X.); gjr19850213@163.com (J.G.); zhaoqiankaisteam@126.com (Y.Z.); wl20008@sina.com (L.W.); 2 Jiangsu Academy of Agricultural Sciences, Nanjing 210014, China; lyd0527@126.com; 3 State Key Laboratory of Systematic and Evolutionary Botany (LSEB), Institute of Botany, the Chinese Academy of Sciences, Beijing 100093, China

**Keywords:** chloroplast, SNP, InDel, high throughput, genotyping, maize

## Abstract

Maize(*Zea mays.* L) is a globally important crop, and understanding its genetic diversity is crucial for plant breeding phylogenetic analyses and comparative genetics. While nuclear markers have been extensively used for mapping agriculturally important genes, they are limited in recognizing characteristics, such as cytoplasmic male sterility and reciprocal cross hybrids. In this study, we performed next-generation sequencing of 176samples, and the maize cultivars represented five distinct groups. A total of 89 single nucleotide polymorphisms (SNPs) and 11 insertion/deletion polymorphisms (InDels) were identified. To enable high-throughput detection, we successfully amplified and confirmed 49 SNP and InDel markers, which were defined as a Varietal Chloroplast Panel (VCP) using the Kompetitive Allele Specific PCR (KASP). The specific markers provided a valuable tool for identifying chloroplast groups. The verification experiment, focusing on the identification of reciprocal cross hybrids and cytoplasmic male sterility hybrids, demonstrated the significant advantages of VCP markers in maternal inheritance characterization. Furthermore, only a small subset of these markers is needed to provide useful information, showcasing the effectiveness of these markers in elucidating the artificial selection process of elite maize lines.

## 1. Introduction

Maize (*Z. mays.* L) is a traditionally cultivated and highly commercialized crop with diverse applications in food, fruit, feed, and fuel. It is currently the most significant crop in terms of planting areas and production worldwide and in China [1,2]. With the rapid development of crop breeding, the number of maize germplasm resources and hybrid cultivars has been increasing annually [3]. Molecular markers play a crucial role not only in understanding the impact of artificial selection but also in accelerating breeding programs and ensuring the authenticity and distinctiveness of germplasm resources and commercial varieties for seed quality control [4,5].

Several types of molecular markers targeting the nuclear genome have been developed and applied for maize variety identification, including simple sequence repeats (SSRs, also known as microsatellites) [5,6], single-nucleotide polymorphisms (SNPs) [7,8], and insertion/deletion polymorphisms (InDels), which are generally distributed throughout the nuclear genome and exhibit numerous variations. At the same time, the application of nuclear molecular markers has played an increasingly more important role in the construction of fingerprinting databases, germplasm resource analysis, and variety identification of maize. However, traditional nuclear genome markers still have limitations in identifying cytoplasmic male sterility (CMS) and maternal lines of reciprocal cross hybrids. Chloroplast markers serve as ideal complementary genetic tools compare to nuclear molecular markers.

Chloroplasts, which are found in the cytoplasm and play a vital role in photosynthesis, provide energy for plant growth and development but possess a significantly smaller genome size compared with the nuclear genome [9]. They have stable and conserved molecular structures, in most cases basically include single copies of genes (except in some complicated inverted repeats regions), and typically undergo uniparental inheritance in angiosperms [10,11]. However, until now, the cytoplasm genome has not been effectively used. In fact, the chloroplast genome (cpgenome) contains valuable information for maternal inheritance [12], enabling the traceability of maternal genealogy. Additionally, the chloroplast genotype is homozygous, enabling quick, automated, and accurate genotyping on high-throughput platforms.

More recently, the value of the cpgenomes and their molecular markers has been confirmed through various studies leveraging next-generation sequencing (NGS) technologies. However, most studies of cpgenomes have primarily focused on phylogenies, population structures, and geographical origins, using chloroplast barcoding technology and SSR and SNP markers [13,14,15]; this has also become an important means for evaluating crop germplasm resources and researching crop domestication [16,17]. For example, chloroplast molecular markers were developed on rice and jujube for species identification and breeding [18,19]. The application of SNP and SSR markers in crops has mainly been employed for interspecific identification rather than intraspecific identification, which was archetypal [20,21]. For example, Shiikuwasha cpgenomes [22] were sequenced to discover the SNP loci in cpgenomes and for the development of accurate markers [23], which represent one of the few archetypes of variety identification.

In this study, we performed next-generation sequencing and conducted a comparative analysis of the cpgenomes of 176 samples, encompassing 168 representative maize cultivars, 7 teosintes, and 1 outgroup material. Additionally, we successfully developed and validated the Varietal Chloroplast Panel (VCP) using the KASP assay, which can be effectively utilized for maize variety identification. At the same time, compared with traditional approaches such as endosperm detection or fertility recovery tests, we developed a much simpler experimental method for identifying reciprocal cross hybrids and cytoplasmic male sterility hybrids of maize, leveraging the significant advantages of VCPs in maternal inheritance characterization. Our research lays the groundwork for constructing large-scale chloroplast DNA fingerprint libraries, enabling the traceability of parental sources and the distinction of maize varieties.

## 2. Material and Methods

### 2.1. Sample Collection

In total, 176 samples were collected representing an outgroup of *Tripsacum dactyloides* (L.) and a sub-species of the genus *Z.*. Within *Z.* genus, we sampled representative wild species and three wild forms of *Z. mays*. The majority of the samples consisted of breeding materials or inbred lines of maize cultivars, such as common maize, waxy maize, sweet maize, popcorn maize, etc., which are cultivated across China. These breeding lines encompassed hybrids, mutants, and doubled haploids. Additionally, the C-, S-, and T-cytoplasmic male sterile (CMS) lines [24], cross-incompatible lines, and inducer lines were also included (Table S1).

Thirteen trios contained 13 pairs of parents and hybrids (Table S2), three reciprocal cross lines contained three pairs of parents and their hybrids/reciprocal cross hybrids, and three male sterility lines contained three pairs of parents and hybrids in which the father was sterile.

### 2.2. DNA Extraction, Library Construction, and Sequencing

Ten seeds of each sample were grown at 25 °C with light for seven days. Subsequently, young leaves were selected and ground into a fine powder using liquid nitrogen. The total genomic DNA was extracted from the pooled leaves (~2.0 g) using the CTAB procedure [25]. The concentration of the extracted DNA was quantified using a NanoDrop 2000 spectrophotometer (Thermo Scientific, Boston, MA, USA) and was adjusted to approximately 20 ng/μL for PCR amplification. The genomic DNA was fragmented into smaller fragments of 500 bp using ultrasonic technology. The DNA libraries were constructed using an NEBNext^®^ DNA Library Prep Set and sequenced on an Illumia HiSeq 4000 platform with the PE150 model. The clean data for each sample exceeded 10 GB, and the percentage of the Q30 base exceeded 75%.

### 2.3. Chloroplast Genome Assembly and Annotation

Quality control of the raw reads was performed using fastp (version 0.20.1). Then, the clean reads were assembled into contigs using SOAPdenovo2 [26] and SPAdes [27]. Contigs corresponding to the cpgenomes were identified by performing a BLASTn search against the reference cpgenomes [28]. Finally, these contigs were assembled into complete genomes using Sequencher 4.10. The reliability of the resulting genomes was confirmed by independently mapping all reads back to the new genomes respectively using Geneious 8.1 [29].

The newly generated cpgenomes were annotated using DOGMA (Dual Organellar GenoMe Annotator) [30], employing BLASTX and BLASTN to identify the positions of the encoding genes and the RNA genes. The promoters, terminators, and exon–intron boundaries of potential encoding genes were determined through this annotation process. For genes such as *rps16*, *petB*, and *petD*, where the exon–intron boundaries were not accurately predicted by the annotations, we referred to the published genomes of closely related species. The genome structure maps were generated using OrganellarGenomeDRAW [31,32].

### 2.4. Phylogenetic Analyses

The 176 newly generated cpgenomes and 14 publicly obtained sequences were subjected to multi-sequence alignment using MAFFT [33] and manually adjusted using Se-Al. Phylogenetic analyses were conducted using the following methods: the maximum parsimony (MP) method with PAUP* 4.0 b10, the maximum likelihood (ML) method with RAxML 7.04, and Bayesian inference with MrBayes 3.2.2 [34]. The second copy of the inverse repeat (IR) region was not removed, as the IR region evolves more slowly than the large single-copy (LSC) and small single-copy (SSC) regions. Containing both copies was considered equivalent to weighting the IR region twice. Gaps were treated as missing data. MP analyses employed heuristic searches with 100 random sequence additions and tree bisection reconnection (TBR). The robustness of the topology was assessed through bootstrap analysis using 1000 replicates.

The substitution model for the ML BI analyses was determined using jModelTest [35] without partitioning. The robustness of the topological structure was evaluated using bootstrap analysis with 1000 replicates. In the BI analysis, the Markov chain Monte Carlo (MCMC) ran for 100 million generations, sampling one tree every 10 thousand generations. The first 20% of the trees was discarded as burn-in. The SNP haplotype data were used to build a NeighborNet network based on the uncorrected p distance using SplitsTree 4 [36] with 1000 bootstraps.

### 2.5. Development and Validation of Maize Chloroplast Markers Using the KASP Assay

A hundred maize chloroplast SNP and InDel loci from 176 samples and 13 trios were used to design primers for the KASP assay (LGC Genomics, Teddington, UK). The primers were designed based on the 60 bp conserved flanking sequences of the variation loci. KASP assays were performed in a 1 μL reaction system, which included 30 ng total genomic DNA, 0.5 μL of 2× KASP ROX standard reaction mix (Kbiosciences, Herts, UK), 0.014 μL assay mix (12 µmol/L each allele-specific forward primer and 30 µmol/L reverse primer), and 0.5 μL distilled water. The PCR was carried out on a Hydrocycler (HC-64) (LGC Genomics, Teddington, UK) under KASP standard touchdown cycling conditions: 94 °C for 15 min, followed by ten cycles of touchdown PCR from 61 °C to 55 °C (decreasing by 0.6 °C each cycle), followed by 30 cycles of 94 °C for 20 s and 58 °C for 1 min. PCR fluorescent endpoint readings were obtained using the BMG Pherastar (LGC, Middlesex, UK), and the cluster calls were visualized using Klustercaller software 4.1.1.23135 (LGC, Middlesex, UK).

### 2.6. Development and Verification of Intraspecific Loci

The genotyping data obtained from the KASP assay for the eligible markers were utilized to calculate the *F*_st_ value using the ‘weir-f_st_-pop’ function of VCF tools. *F*_st_ values exceeding 0.9 were considered indicative of a correlation between the specific loci and the corresponding chloroplast types. For validation purposes, primers for the KASP assay were designed for three reciprocal cross lines and three male sterility lines. These were performed to confirm the consistency of the eligible markers with maternal inheritance.

## 3. Results

### 3.1. Annotation and Analysis of the Chloroplast Genome of Maize

**Figure 1:**
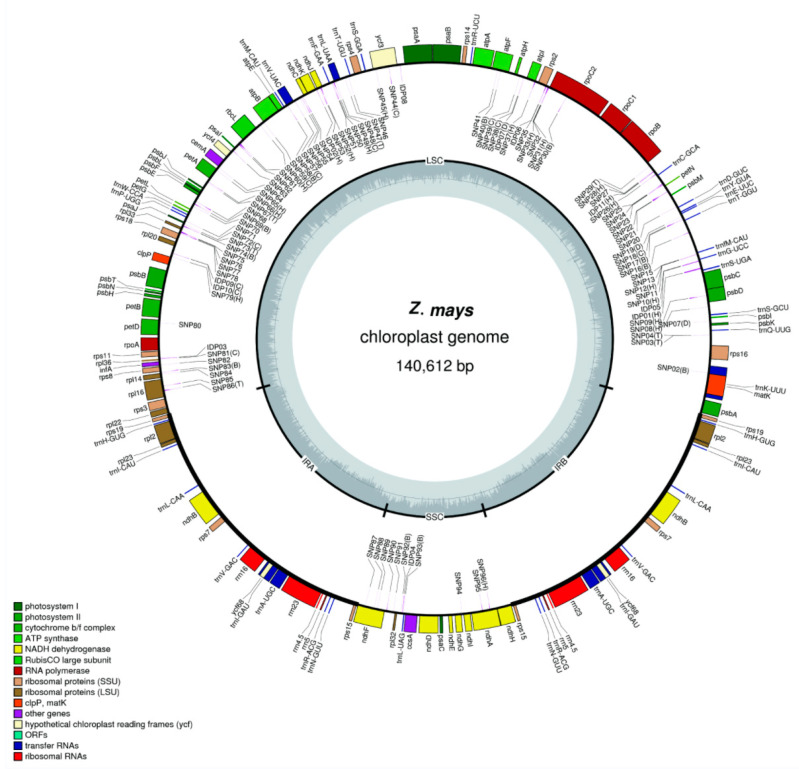
A representative scheme of 176 chloroplast genomes of *Z.* and the distribution of variations (SNPs and InDels) across the chloroplast genome map of diverse *Z. mays* species. The chloroplast-specific loci are marked in brackets. The inner circle delineates the inverted repeat regions (IRa and IRb), the small single-copy region (SSC), and the large single-copy region (LSC). Functional categories of genes are color-coded.

A total of 176 cpgenomes were assembled, ranging in size from 140,440 to 140,810 bp, with an average GC content of 38.4%. Each cpgenome had two reciprocally inverted repeats (IRa and IRb), one in the SSC region and one in the LSC region (Figure 1, Table 1 and Table S3). Within each cpgenome, there were 110 unique genes, including 77 protein-coding genes, 29 tRNA genes, and 4 rRNA genes. Among these genes, 62 protein-coding and 24 tRNA genes were located in the LSC region, 11 protein-coding genes and 1 tRNA gene were located in the SSC region, and 4 protein-coding genes, 4 tRNA genes, and 4 rRNA genes were located in the IR region.

Pairwise comparisons of the cpgenomes among all the lines revealed a total of 100 loci variations, including 89 SNP variations and 11 InDel variations (Table S4). It was observed that the InDel variations were significantly less common than the SNP variations in the maize cpgenomes. Among these variations, 75 occurred in the intergenic region, which consisted of 66 SNPs and 9 InDels, while 23 variations were located in the genic region, all of which were SNPs. The average density of these variations was approximately 1 in every 714 bp across all the maize cpgenomes, indicating a high level of conservation within the cpgenomes of the same species compared with different species. The most conserved regions of the maize cpgenomes were *psa*, *psb*, *rpo*, *rbcL*, *rbcS,* and *trn*, whereas the most variable regions were *atp*, *trnS-trnf*, *ndh*, *rps,* and *rpl*.

### 3.2. Typing Maize Varieties Based on the Chloroplast Genome

**Figure 2:**
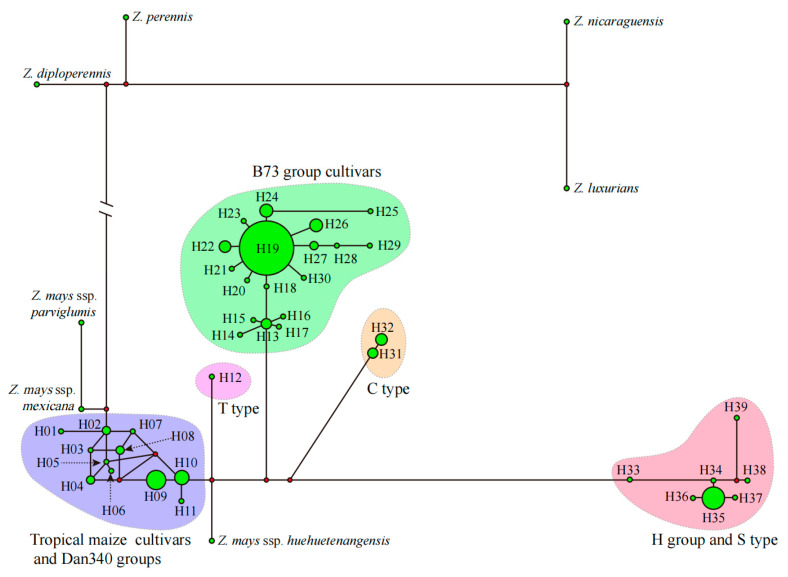
Maximum likelihood tree together with branches resolved using maximum parsimony and Bayesian inference methods, as indicated by bootstrap and post probability values. Five groups were resolved in *Z.* sect. *Z.* Note that the long branches of three genomes from GenBank were shortened, and the names of cultivars were omitted for presentation on one page.

After combining 14 cpgenomes downloaded from GenBank, the dataset comprised a total of 190 genomes with an aligned length of 141,765 sites. Within *Z. mays*, five major groups were identified (Figure 2). Group C exclusively consisted of C-CMS, while Group H comprised S-CMS and Huanggai cultivar groups specific to China. Group T consisted of T-CMS. GroupD referred to the tropical cultivars and Dan340 cultivar groups specific to China, along with three subspecies: subsp. *huehuetenangensis*, subsp. *mexicana*, and subsp. *parviglumis*. Group B included exclusive cultivars of subsp. *mays.* All five groups showed significant divergence when compared with the wild species in sect. *Z. perennis* and *Z. diploperennis* exhibited a close relationship (Figure 2), while the genetic divergence between *Z. luxurians* and *Z. nicaraguensis* appeared to be less notable based on their branch lengths.

### 3.3. Development and Validation of Maize Chloroplast Markers

In order to validate the variations in maize cpgenomes and develop a Varietal Chloroplast Panel (VCP) for maize variety identification, we designed assays containing 100 variation loci obtained from the chloroplast sequencing results on the KASP platform. Four factors were considered in marker selection: 1. the fluorescent signals of the primers were observed and showed a distinct cluster, indicating significant similarity in fluorescence signals; 2. rare fluorescent signals were detected in the no-template controls, indicating the absence of primer dimers and false positive results; 3. the genotype data obtained from KASP assays were consistent with the sequencing results; 4. the genotype data reflected the maternal genetic characteristics in the triad, reciprocal cross, and other samples. The results showed that 59 pairs of primers were successfully amplified on the KASP platform, including 56 SNPs and 3 InDel markers (Table 2). At the same time, 38 loci with rare alleles and minor allele frequencies of less than 1% were eliminated. The genotype data obtained from both the sequencing and KASP platforms were found to be 100% consistent, and the 59 loci demonstrated maternal genetic characteristics associated with chloroplast markers (Tables S5 and S6).

### 3.4. Selection of Maize Chloroplast-Specific Loci

**Figure 3:**
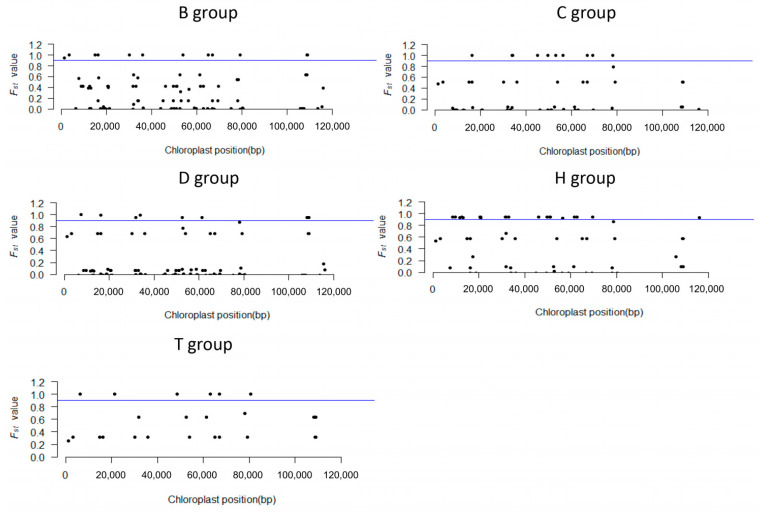
Selection of chloroplast-specific type loci. The F_st_ value of the maize chloroplast genome was calculated for this selection of chloroplast-specific loci.

**Figure 4:**
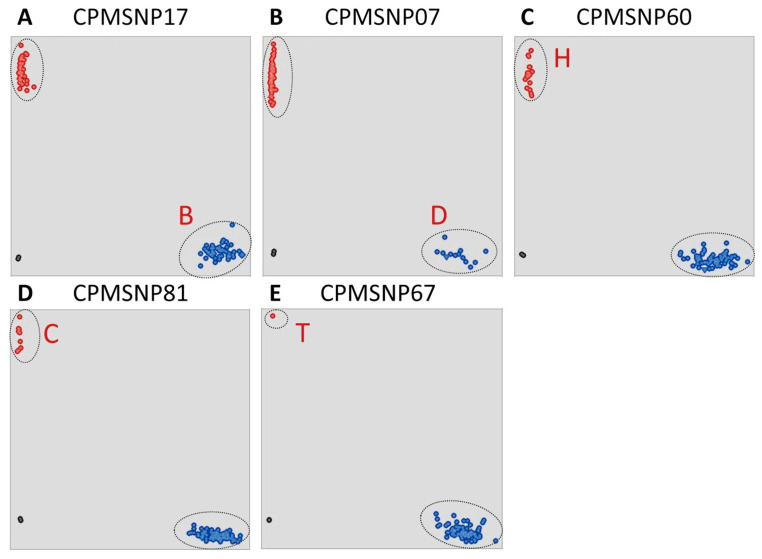
Genotyping of maize VCP markers visualized using the KASP assay. (**A**) The SNP17 loci of group B. (**B**) The SNP07 loci of group D. (**C**) The SNP60 loci of group H. (**D**) The SNP81 loci of group C. (**E**) The SNP67 loci of group T.

To distinguish the five maize cpgenome groups, we analyzed the association between the various loci and chloroplast types. Based on F-statistics, 49 loci showed significant correlations with each specific chloroplast group (Figure 3), ranging from 3 loci for the D group to 19 loci for the H group. The remaining 10 loci were not specifically associated with the five chloroplast groups (Table 2). Among them, five loci—CPMSNP17, CPMSNP07, CPMSNP60, CPMSNP81, and CPMSNP67—were selected as the core chloroplast loci set for differentiating the five chloroplast types. High-throughput genotyping, using the KASP platform, confirmed the excellent experimental genotyping results, demonstrating the high polymorphism of these five loci among the tested lines and their potential for analyzing germplasm recourses (Figure 4).

### 3.5. VCP Validation Using a Maternal Lineage Tracing Experiment

**Figure 5:**
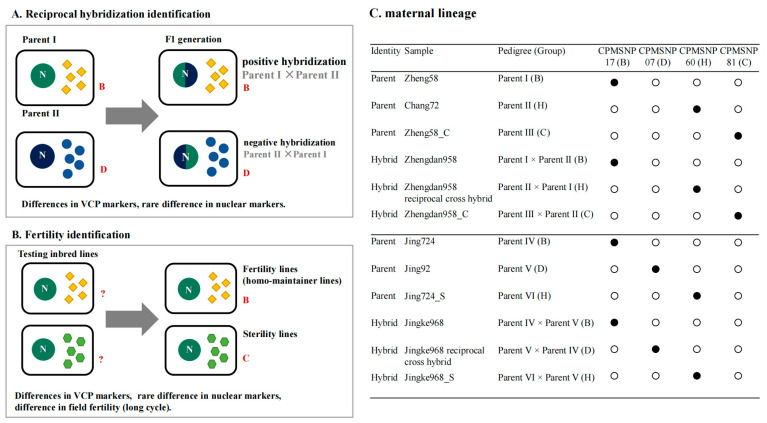
VCP markers using reciprocal hybridization, fertility identification, and the maternal lineage feature. (**A**) Reciprocal hybridization identification. (**B**) Fertility identification. (**C**) Maternal lineage.

Furthermore, to validate the VCP and trace maternal lineages, we conducted a maternal lineage tracing experiment using the KASP platform. By designing primers for the hybrid progeny sites, we demonstrated that the five selected VCP loci had the ability to accurately trace the maternal lineage of three reciprocal cross lines and three male sterility lines. Each hybrid maize offspring had exactly the same genotype as its female parent (Figure 5).

## 4. Discussion

A total of 89 SNP and 11 InDel variations were identified in the maize cpgenomes through analysis of high-throughput sequencing data. Among them, 59 variations were successfully amplified using KASP primers, and they exhibited maternal inheritance. Forty-nine VCP markers played a crucial role in identifying the five distinct groups observed in the maize cpgenomes. Furthermore, these markers showed significant advantages in maternal lineage tracing and provided valuable complementary information to nuclear markers for identifying CMS materials and reciprocal cross hybrids.

Traditionally, the detection of variation loci in the cpgenomes was limited by the available technologies, leading to the development of chloroplast SSR markers using PCR amplification with highly conserved cpgenome sequences. However, with the advent of new high-throughput sequencing technologies and advanced analysis tools, it has become easier to discover SNP loci. The accuracy of the variations, including both the variations themselves and their flanking sequences, is crucial. In this study, the genotyping results of the developed markers demonstrated a high success rate of 95%. Notably, the IDP01 marker represented a large InDel and was previously reported in a study using agarose electrophoresis [37]. Although a rigorous screening method was utilized to identify variations, it was observed that one variation was initially recognized as two InDel loci, CPMIDP09 and CPMIDP10. These loci have now been merged into a single marker for further utilization.

Reciprocal crosses were shown to impact various maize traits, such as grain yield [38] and kernel sink capacity [39]. Additionally, there are noticeable differences in corn grain yield between temperate and tropical races [40]. However, tracing maternal genealogical data using nuclear markers to identify reciprocal cross-hybrids has proven challenging. Existing solutions involve detecting endosperm using two copies of the maternal information or analyzing pericarp tissue in the hybrids [41]. Both methods require complex procedures and rely on seeds as the original material, limiting their applicability. In contrast, chloroplast markers offer a straightforward means of distinguishing reciprocal cross hybrids, as long as both parents belong to different chloroplast groups. For example, Huangzaosi improved lines group, the germplasm widely used in China, and the Reid group, which exhibits significant differences in chloroplast loci genotypes, can be easily distinguished using our technology.

In this study, specific loci from each chloroplast group were employed to identify CMS hybrids, a task that was previously challenging using traditional methods such as fertility recovery tests and Southern blotting. CMS varieties differed from the original fertile variety in the cpgenomes, while they maintained identical nuclear genomes. They can be regarded as a special type of EDV that may see increased cultivation in the future. By utilizing only five markers, the five chloroplast groups can be distinguished, and this number can be reduced to three when the material is known as fertile or sterile. High-throughput genotyping platforms decrease the cost and time requirements by more than half, providing efficient and accurate genotyping of markers. For example, analyzing 10,000 samples using five specific loci would take approximately 2 h.

Moreover, some of these loci may be specific to sub-groups within the known pedigree, while others may be variety-specific loci that offer more precise maternal group information. However, as the number of maize varieties increases, the specificity of variety-specific loci may decrease; yet, they will still represent rare allele genotypes. Overall, expanding the sampling range necessitates the consideration of additional combinations of highly polymorphic loci to distinguish groups within the species.

Establishing a chloroplast DNA fingerprinting database based on the core set of chloroplast-specific loci will further enhance molecular-level information regarding massive germplasm resources and cultivar varieties, especially on high-density chip platforms. The chloroplast loci identified in this study have also been incorporated into the Maize6H-60K array, which is already a commercially available chip, providing valuable molecular information. In light of a highly stable structure, the maternal genetic characteristics of the cytoplasmic genome, and large differences in the nuclear genome structures of cultivars of maize, cluster analysis of nuclear and cytoplasmic genomes may provide a new perspective for the origin and evolution of maize, tracing of the genome of elite inbred lines, and the exchange of breeding selection of maize germplasm.

## Figures and Tables

**Table 1:** Summary of the chloroplast genome of *T. dactyloides* and 176 chloroplast genomes of *Z.* genus.

	*T. dactyloides*	*Z.* genus
Size(bp)	140,982	140,440–140,810
LSC length (bp)	82,928	82,391–82,741
IR length (bp)	22,750	22,737–22,771
SSC length (bp)	12,554	12,527–12,546
Total number of genes	110	110
Protein coding genes	77	77
tRNA	29	29
rRNA	4	4
% GC content	38.40%	38.40%

**Table 2:** Information on the VCP markers obtained using the KASP assay.

Marker	Variation Loci	AlleleFAM	AlleleHEX	Specific Type	Corresponding Alleles of cpGenome Groups	Primer_AlleleFAM	Primer_AlleleHEX	Primer_Common	Flank1	Flank2
CSNP01K	CPMSNP01	T	G	B	T	AGCAATCTGAGTTTTTCATTTTTACTAACTTA	GCAATCTGAGTTTTTCATTTTTACTAACTTC	CTTCATTTACCAAATCCAAAAATTTGGGAA		
CSNP02K	CPMSNP02	A	G	B	A	AACAAACATAAACTAATTAGATAGAAAAGGAGT	CAAACATAAACTAATTAGATAGAAAAGGAGC	GAAAGAAAGGGAGTCTAATCCATAGAACTT		
CSNP03K	CPMSNP03	C	G	T	G	AGGATCCATTTGACCCCCAATATG	AGGATCCATTTGACCCCCAATATC	GGAAAATAAATAGGGGGTACTTCTTTTCTT		
CSNP04K	CPMSNP04	A	G	T	G	AAATAAATAGGGGGTACTTCTTTTCTTTCA	AAATAGGGGGTACTTCTTTTCTTTCG	CTTACAGGATCCATTTGACCCCCAA		
CSNP07K	CPMSNP07	T	G	D	T	GCAGGGGGTAGAAAGGCTGATA	CAGGGGGTAGAAAGGCTGATC	CTACATTGAATGTATAGCTGCAGCAATAAA		
CSNP08K	CPMSNP08	C	A	H	A	AATAAATAAAGGGTTTCAAAAGTCAATTTTTC	AATAAATAAAGGGTTTCAAAAGTCAATTTTTA	GGAATTCTGAAAAAAAAAAGAAAGATATTG		
CSNP09K	CPMSNP09	C	T	H	T	TCAACGTCCAATTATGAAATCCTTGG	GTTCAACGTCCAATTATGAAATCCTTGA	GTAGCAGCTATATTTCGGTTCATCCTTT		
CSNP10K	CPMSNP10	C	A	H	A	ATATTTTATAGGGTATATCCACCTGG	CCTATATTTTATAGGGTATATCCACCTGT	ACATAGACGGTCGACCCAGACATA		
CSNP12K	CPMSNP12	T	C	H	C	TTTCTTTCATTTTTTTTTTTTTTTTTTCT	GCTTTTCTTTCATTTTTTTTTTTTTTTTTTCC	TATCCAACCCTTTTTTTTTATTTAGCAGGC		
CSNP16K	CPMSNP16	A	G	B	A	ATGTAGGATATGCTTTTTATTTTTTGTTGGA	GTAGGATATGCTTTTTATTTTTTGTTGGG	CTGCAGAGTATCAAAATTATACTACTGCCT		
CSNP17K	CPMSNP17	C	T	B	C	AAATTCATTCATTTCTTTTTTGAAAATGTCC	CTAAATTCATTCATTTCTTTTTTGAAAATGTCT	GGCATCTCGCACTAAACTAAGTCATAAA		
CSNP18K	CPMSNP18	T	G	C	G	GTGCTCGTTTAGTGTTCAGACCA	GTGCTCGTTTAGTGTTCAGACCC	CTTAGTTTAGTGCGAGATGCCCACAT		
CSNP19K	CPMSNP19	A	C	D	C	AGTTGATGGTTAGGTTAATTCACGGAT	GTTGATGGTTAGGTTAATTCACGGAG	TAACCTTAAAAAGCTTAAAAAGTAGGGGAT		
CSNP21K	CPMSNP21	A	C	/	/	GGTTTTTTCCTTTTACTTTTTTTCTTTTACTAT	GGTTTTTTCCTTTTACTTTTTTTCTTTTACTAG	GAGAAAAATAATACGAGAATAGACTAGAAT		
CSNP22K	CPMSNP22	A	G	/	/	CCTTTTTTAAGCATGAAAGATTCGTAGGT	CTTTTTTAAGCATGAAAGATTCGTAGGC	CGAGAATAGACTAGAATAGATTATAGTAAA		
CSNP26K	CPMSNP26	A	G	H	G	ACTTACTTTTTTAGAATCTTTTTCAAAAAATA	ACTTACTTTTTTAGAATCTTTTTCAAAAAATG	AGCGAAACTGGATCCAAAAAAGCAGAAAT		
CSNP28K	CPMSNP28	T	G	H	G	ATTTATTCTTATTCTATTTTATTATGCCATTCA	TATTCTTATTCTATTTTATTATGCCATTCC	TCTTAAATCGGTATTCCCCCCCATTATTT		
CSNP29K	CPMSNP29	G	T	T	T	ATATTCTAAAAAGATTGGATAGCAAAGATTTC	GATATTCTAAAAAGATTGGATAGCAAAGATTTA	GCTTTATCCCGTTTCATAGAAAGGAGATA		
CSNP30K	CPMSNP30	A	G	B	A	TAGGAAATCGCGAATTAGATCATTTGTTT	GGAAATCGCGAATTAGATCATTTGTTC	GCTCGTGCTTCTCTTGTTGAGGTAA		
CSNP31K	CPMSNP31	T	G	H	G	TTAAGTATACATAAAGCAATTTTTTTTACTTT	TAAGTATACATAAAGCAATTTTTTTTACTTG	GTTAGCATTCTAAGGTCAAAAGTATAGTTT		
CSNP33K	CPMSNP33	T	G	H	G	ACTGACTTCTTTTACTTATTAAAATACAATTTA	ACTGACTTCTTTTACTTATTAAAATACAATTTC	CTAACAGGTCTGATTTTCGATTTTGTACTT		
CSNP37K	CPMSNP37	C	T	H	T	CAATTTTTATCAGAGGACAATATGAATATTAC	CAATTTTTATCAGAGGACAATATGAATATTAT	TATAACCCCTTGAGTGTTTTAATGGAACAT		
CSNP38K	CPMSNP38	G	A	C	A	ATTCTAAAATCATTCTTTAGAAAGCCACAC	CTAAAATCATTCTTTAGAAAGCCACAT	GGCCAAGTCAGGTTAGATCTATATCTTTA		
CSNP39K	CPMSNP39	A	C	C	C	ATGGGAACTCAAAGATATCGAAGAGTA	GGGAACTCAAAGATATCGAAGAGTC	CAACCAATCACTCTTTTATTCCATCCTTTT		
CSNP40K	CPMSNP40	A	T	B	A	CTATCAATTTTTATTTTCCATTTATTTAGTTA	CTATCAATTTTTATTTTCCATTTATTTAGTTT	GTTTCTTTATTTGTGTTTGCTCTGTTAGTT		
CSNP41K	CPMSNP41	A	T	/	/	TTATGATCTCTTCCCGAACCAAACAT	TATGATCTCTTCCCGAACCAAACAA	CGGGAGAGCCAAATGAATCGAAAGAT		
CSNP44K	CPMSNP44	A	C	C	C	GCCTATACTACTATTCTATGGATAAAGCT	CCTATACTACTATTCTATGGATAAAGCG	TCGCTCACTAATTGATCTTTACGGTGTTT		
CSNP45K	CPMSNP45	T	G	H	G	AAGCGCGGGTTTCCTTTACTAATTTT	AGCGCGGGTTTCCTTTACTAATTTG	AGAGAGAGGGTTCGCATAGAGAGAA		
CSNP47K	CPMSNP47	T	G	T	G	GAACTATTTATCCTTAAATTATTAACAAATAA	GAACTATTTATCCTTAAATTATTAACAAATAC	GCCAAGAGATTGGCATTTTCATTTGATCAT		
CSNP48K	CPMSNP48	T	A	C	A	ATCCTCGTCCGATTAATCCACTTTTA	ATCCTCGTCCGATTAATCCACTTTTT	CCTTCAATTCATTGTTTTCGAGATCTTTTA		
CSNP49K	CPMSNP49	T	G	H	G	AGTGAATCTTAAACCCATTGATAAAAGA	AGTGAATCTTAAACCCATTGATAAAAGC	TTTATTCCCTAACCATAGTTGTTATCCTTT		
CSNP52K	CPMSNP52	C	T	H	T	CCAAAAGGATAATCCTAGAATCCCG	CCCAAAAGGATAATCCTAGAATCCCA	ATCGGCACTTCTCCAAACCCAGAAA		
CSNP56K	CPMSNP56	A	C	/	/	CCTATTTTAATATATATTAATCATCCTATTTT	CCTATTTTAATATATATTAATCATCCTATTTG	ACTTACTACTAATTGGATTAGAACCTAATT		
CSNP57K	CPMSNP57	G	A	C	A	TATTTAGTACTTGTTTATAGACTCGAC	CCTTATTTAGTACTTGTTTATAGACTCGAT	AATGCTTTTATCTCTATTCTATGGCGCAAT		
CSNP58K	CPMSNP58	A	T	B	A	CAACAAGGTCAATTATGTTCATTGCATAAA	CAACAAGGTCAATTATGTTCATTGCATAAT	GCGCCAATGCTTTTCAAGGGAACTT		
CSNP59K	CPMSNP59	G	A	C	A	ATTCAACAAGAAAAAAAATTTCGACAAATTCC	ATTCAACAAGAAAAAAAATTTCGACAAATTCT	GCGAAGTAGTAGGATTGGTTCTCATAATT		
CSNP60K	CPMSNP60	A	T	H	T	GATTCAAAATATCAAAGGGGAAGAACTTTA	CAAAATATCAAAGGGGAAGAACTTTT	GCAACCCAAACCCTAATCTTTATTTTACAA		
CSNP62K	CPMSNP62	T	G	/	/	AAAGAAATACCTCTTTCAGAATACCCTTTA	GAAATACCTCTTTCAGAATACCCTTTC	CAACTGGGTATTCTATTCCACTTCTACTT		
CSNP64K	CPMSNP64	A	G	/	/	AATTAGCATATTTCTTTTCTTCCTTTAGAAATA	AATTAGCATATTTCTTTTCTTCCTTTAGAAATG	ATTTTGTTAAAAAGGAAAAGGGCTTTCTTT		
CSNP65K	CPMSNP65	G	A	H	A	CGATTTCTGTATCGATCATGATATACG	ATCGATTTCTGTATCGATCATGATATACA	GATATGCGTTTGAAATAGATGTGCGAGTT		
CSNP66K	CPMSNP66	C	T	H	T	TATTTGTTTTGTCAAAGATTACTATTTATTC	CTTATTTGTTTTGTCAAAGATTACTATTTATTT	GGAAGTCCAAAAGACAGACCCGAAT		
CSNP67K	CPMSNP67	A	C	T	C	AGTTGAACTTAATTCAAAAAGTAAAGCAATTCT	GTTGAACTTAATTCAAAAAGTAAAGCAATTCG	CGGGGACACATTTCTTGTGAGCAAA		
CSNP69K	CPMSNP69	T	A	B	T	CCCCTCAAAAAGGGAACTATTCCTA	CCCCTCAAAAAGGGAACTATTCCTT	CCACTTTTGTTGGGGTTCAAAAAACGAAT		
CSNP72K	CPMSNP72	T	C	C	C	TCATATACTAAAAAAGAATTCAAAAAGGGGA	CATATACTAAAAAAGAATTCAAAAAGGGGG	GAGATAGAATTCTTCGTGACATGACGAAA		
CSNP73K	CPMSNP73	T	C	T	C	ATTTCAAAAATTTTGTATTCTATTGGATTGGAT	TCAAAAATTTTGTATTCTATTGGATTGGAC	TTTGTTGTAATTCTTCGAATTCTCGAACAA		
CSNP74K	CPMSNP74	T	G	B	T	TGTATTCTATTGGATTGGATTTGTTCGAT	GTATTCTATTGGATTGGATTTGTTCGAG	TCTAAAGATTTTGTTGTAATTCTTCGAATT		
CSNP79K	CPMSNP79	G	T	H	T	GTATTTCTATTTTCTATAGCATAAAACCCG	AAGTATTTCTATTTTCTATAGCATAAAACCCT	GGATTTCTTGTAAATTTATCTCAAACCTAA		
CSNP81K	CPMSNP81	G	A	C	A	AGGCGTGGGCGAATTAGAGTC	CAGGCGTGGGCGAATTAGAGTT	GTCTTTGTTTATGCTTCGGATTGGAACAA		
CSNP83K	CPMSNP83	T	A	B	T	TAGTAGATTTTGTCTCACGTATATGCTTA	AGTAGATTTTGTCTCACGTATATGCTTT	CATGTTTTCCCTTTTCTTTAAATTTAGGAT		
CSNP85K	CPMSNP85	G	T	/	/	TTTTCTTTTTTAAGTTTAAGAAAGTCAAAATC	TTTCTTTTTTAAGTTTAAGAAAGTCAAAATA	CACATCAATATATAATAGAAAAAGTTAGGT		
CSNP86K	CPMSNP86	A	C	T	C	TTGAATCCTGCAATGGAGCTTCCA	GAATCCTGCAATGGAGCTTCCC	GCAGCCGGGTTAATAAAACTGAGAAAATT		
CSNP91K	CPMSNP91	A	C	/	/	ATACTGAAAGATACTGAAAGATACTTAAATTCT	CTGAAAGATACTGAAAGATACTTAAATTCG	CCACATTAGACAAAATGAACTAAAGAAGAA		
CSNP92K	CPMSNP92	C	T	B	C	CTTGCAATAGGACTTACAACCTCC	CTTGCAATAGGACTTACAACCTCT	CCCATTTATATGGGAATTTTGGATAAGATT		
CSNP93K	CPMSNP93	T	A	B	T	CCAATTTCACCATGGCGGCTAATTTA	CCAATTTCACCATGGCGGCTAATTTT	CCCAGTCTCGACGATTCACGATAAA		
CSNP95K	CPMSNP95	A	G	/	/	AAAAGATATGGAATACAATACAAAAAAGGATCT	AGATATGGAATACAATACAAAAAAGGATCC	GAATAGGGATAAAGGAAGGAAAGAATAAAT		
CSNP96K	CPMSNP96	A	G	H	G	AAAAGATCCTATTTTAACGAATCACACGTA	AGATCCTATTTTAACGAATCACACGTG	TACCATTAACTTTTTGTGTACTAGCAATAT		
CIDP01K	CPMIDP01	ACTGTATACACGGATACAGAATCCGCTATATCCGTTTGTGAAATAAAGGCTAAATCCCCTCCCCTCAACTCCATATCTAAATA	-	H	I	TTTTATTAAAACTTTTTCCTTACCGCTTTTA	CTTTTTATTAAAACTTTTTCCTTACCGCTTTTT		ATGCAAGTCCACTTTCAATATATCTCTGTA	CCCTCCCCTCAACTCCATATCTAAA
CIDP02K	CPMIDP02	-	TCTTT	H	D	CAAGTTTGAAAGATTGTACTGCTCTTTC	GCAAGTTTGAAAGATTGTACTGCTCTTTT	ATTAGGAGGGGTTCTTTTGTGCAGAAAAA		
CIDP04K	CPMIDP04	-	ATGAACTTCTAATG	/	/	AGAATTTAGGAACATTAGAAGTTCATCATTAA	GAATTTAGGAACATTAGAAGTTCATCATTAG	TCTAAAATACAAAATGCATTTCATTGTAG		

## Data Availability

All data generated or analyzed during this study are included in this published article.

## Supplementary Materials

The following supporting information can be downloaded at: https://www.mdpi.com/article/10.3390/genes15030293/s1, Table S1. A list of 176 samples for which chloroplast genomes were determined in the study. Table S2. A list of trio materials used to invalidate the VCP markers. Table S3. Features of 176 complete chloroplast genomes. Table S4. Information on polymorphic chloroplast SNP and InDel loci. Table S5. The typing results of 59 validated loci sequencing data. Table S6. The KASP typing results of 59 validation loci.
